# Improvement and Soil Consistency of Sand–Clay Mixtures Treated with Enzymatic-Induced Carbonate Precipitation

**DOI:** 10.3390/ma14185140

**Published:** 2021-09-07

**Authors:** Yixin Mo, Songlin Yue, Qizhen Zhou, Xiao Liu

**Affiliations:** College of Defence Engineering, Army Engineering University of PLA, Nanjing 210007, China; myx91@foxmail.com (Y.M.); zhouqizhen2016@163.com (Q.Z.); xiao_Liu_z@163.com (X.L.)

**Keywords:** sand–clay mixtures, EICP, permeability, UCS, liquid limit

## Abstract

Recently, microbially induced carbonate precipitation (MICP) has been studied as an alternative for the improvement of sand–clay mixtures. However, the cementing uniformity of MICP-treated sand–clay mixtures cannot be guaranteed. In this present study, enzymatic-induced carbonate precipitation (EICP) was used to deal with it. The ions used in kaolin clay was predicted to affect the production rate for calcium carbonate (CaCO_3_), which was studied using the calcification test. The solidification test was conducted using two different methods (the premixing method and the diffusion method). The permeability, unconfined compressive strength and the content of CaCO_3_ of treated samples were obtained to evaluate the solidification effect of the EICP method. Moreover, in EICP treatment, the particle aggregation decreased the liquid limit, but the addition of solution increased it. Therefore, there were contrary effects to the soil consistency. In this study, the two types of liquid limits of treated samples were measured with deionized water and 2M-NaCl brine, respectively. The results show that the Al_2_O_3_, NaCl and MgCl_2_ in the kaolin clay had a slight impact on the production rate for CaCO_3_, while FeCl_3_ significantly inhibited it. The EICP method can improve sand–clay mixtures and decrease their permeability. Different from MICP, the EICP method can guarantee the uniformity of treated samples. Moreover, the liquid limit of the sample treated with the premixing method decreased, while that of the sample treated with the diffusion method increased firstly and then decreased with the increasing treatment cycles. Different from the deionized water, the pore-fluid chemistry had a larger effect on the liquid limit with 2M-NaCl brine.

## 1. Introduction

In the engineering practices, several materials (e.g., cement, lime) are often injected to improve the strength or stiffness of soils to meet the requirements of the application because the soil particles are cemented together. However, the commonly used materials may cause a negative influence on the environment [1]. Over recent years, a novel alternative method based on induced calcium carbonate (CaCO_3_) precipitation has attracted the extensive attention of researchers in the material engineering field and geotechnical engineering field [2,3,4]. The method was named microbially induced calcium carbonate precipitation (MICP) or enzymatic calcium carbonate precipitation (EICP). In MICP or EICP, the produced acid radical ions can bind with metal ions to form minerals with cementation properties, such as CaCO_3_ [5,6,7]. The obtained CaCO_3_ precipitation forms a bridge between soil particles or fills in the soil pores, eventually leading to strength improvement and permeability reduction [5,8].

Previous studies have demonstrated that the MICP technique can improve the properties of sands and silty sands [9,10,11,12]. There were several studies focused on the sands because the physical properties of sandy soils are easily studied. However, there were only few studies concerning the solidification of sand–clay mixtures. To replace chemical stabilizer, Morales et al. [13] used the MICP technique to produce CaCO_3_ precipitation so as to form clay phyllites. In the study of [14], three different MICP methods were used, and the different treatment effects were evaluated to determine the controlled clay percentages for different methods. The relations between the nutrient solution, bacteria and the soil were investigated by Cardoso et al. [15]; however, the soil they used were mainly sands. Sun et al. [16] used the MICP method to improve sand–clay mixtures and the MICP solidification. In their study, the maximum percentage of clay was only 7.5%, because adding too much clay would affect the void volume, hindering nutrient access to the bacteria and resulting in lack of space for bacterial growth [17]. Compared with the MICP technique, the EICP technique can also produce CaCO_3_ precipitation, but the size of the solubilized urease enzyme was smaller than the that in MICP; therefore, the EICP technique can be used to deal with the soils with smaller particles or pores [18,19]. In this study, the EICP technique was applied to solidify sand–clay mixtures and the mass percentages of clay in soils were relatively larger than the materials used in Sun et al. [16].

There were limited studies in the literature focusing on EICP-solidified sand–clay mixtures. Therefore, EICP was used to produce CaCO_3_ for the improvement of sand–clay mixtures with different mass percentages of clay soils. According to the chemical analysis by Cardoso et al. [15], clays mainly contained silicon (Si) and aluminum (Al); the contents of ferrum (Fe) and sodium (Na) were relatively small. The contents of other elements in clays were quite small; therefore, they can be ignored in this study. The effect of Si on the production rate for CaCO_3_ was also not studied here, because Si is chemically inert. With regard to the compositions the effects of Na and Mg on MICP have been already studied by previous researchers [20,21]. However, there were few previous studies about the effect of the compositions in kaolin clay on EICP. Therefore, the effects of aluminum oxide (Al_2_O_3_), Na, Mg and Fe on the production of CaCO_3_ were studied. The solidification tests were conducted; the permeability, unconfined compressive strength (UCS), and contents of CaCO_3_ were measured to investigate the treatment effects of EICP solidified sand–clay mixtures. Moreover, in EICP treatment, the particle aggregation decreased the liquid limit, but the addition of solution increased it. Therefore, there were contrary effects to the soil consistency. In this study, the two types of liquid limits of treated samples were measured with deionized water and 2M-NaCl brine.

## 2. Materials and Methods

### 2.1. Urease Enzyme

The urease enzyme is an important part of the EICP, which can decompose urea (CO (NH_2_)_2_; Equation (1)) to obtain carbonate ions. CaCO_3_ precipitation can be obtained when the carbonate ions bind with calcium ions (Equation (2)). In nature, the urease enzyme can be found in plants, algae, and some types of bacteria [22]. Previous researchers often used the urease enzyme extracted from jack beans for the application of EICP (Sumner, 1926). The urease enzyme in powder was bought from Sigma Aldrich Company Ltd. (St. Louis, MO, USA) for experiments. To control the initial urease activity, the urease enzyme with an activity of 1030 U/g was used for all tests. In addition, the calcium acetate was used to provide calcium ions.

(1) $$\mathrm{CO}{\left({\mathrm{NH}}_{2}\right)}_{2}+2H_{2}O\overset{\mathrm{urease}\;\mathrm{enzyme}}{\to }2{\mathrm{NH}}_{4}^{+}+{\mathrm{CO}}_{3}^{2-}$$

(2) $${\mathrm{CO}}_{3}^{2-}+{\mathrm{Ca}}^{2+}\to {\mathrm{CaCO}}_{3}$$

### 2.2. Effect of the Ions in Kaolin Clay on Production of CaCO_3_

The components in different clays might be different. To ensure the repeatability of the results, a commercial kaolin clay was used in this study. In the kaolin clay, there were several minerals (kaolin, quartz and muscovite), whose formula are Al_2_O_3_·2SiO_2_·2H_2_O [23]. According to the particle analysis, the percentage of particles with a size smaller than 2 μm reached 90%. The effect of Si on the production rate for CaCO_3_ can be ignored. As for Na and Mg, the effect of them on MICP has been already studied [20,21]. However, there were few previous studies about the effect of the ions in kaolin clay on the production of CaCO_3_ in EICP. Therefore, Al_2_O_3_, Na, Mg and Fe with different contents were added to the urease solution to study it.

Al_2_O_3_ at different weight fractions (0 g, 1 g, 2 g or 3 g) was added to the 100 mL of urease solution to study the effect of Al_2_O_3_ content on the production rate for CaCO_3_. For the production of CaCO_3_, 100 mL of the cementation solution was mixed with the urease solution. The cementation solution was the mixture of 0.75 M of urea and 0.5 M of calcium acetate. After 48 h, the production rate for CaCO_3_ was measured.

In nature, NaCl is a common material. The elements of Mg and Fe are always present in a bivalent from (Mg^2+^) and trivalent form (Fe^3+^), respectively. In the present study, NaCl, MgCl_2_ and FeCl_3_ at different weight fractions (0, 0.5, 1.0, 1.5 and 2.0 g/L) were used to study the effect of them on the production rate for CaCO_3_. Similarly, 100 mL of urease solution was added to 100 mL of cementation solution, and the production rate for CaCO_3_ was measured after 48 h.

The tests were conducted in fluid with a temperature of 30 °C and initial pH of 8.0 in the test tube. The actual produced amount of CaCO_3_ was measured via the acid washing method, described in Sun et al. [24].

### 2.3. EICP Sand–Clay Mixtures Solidification in PVC Cylinders

#### 2.3.1. Sand–Clay Mixture Preparations

Sands from the Yangtze River were used for the solidification test in polyvinyl chloride (PVC) cylinders with an inner diameter of 4.5 cm and height of 10.0 cm. The sand had poor gradation. The sands with a median diameter (D_50_) of 0.3 mm are classified as SP based on the USCS classification system. In this study, the sand–clay mixtures were divided into four groups because of different mass percentages of kaolin clay added: 2.5%, 5%, 7.5% and 10%. In addition, a sample without clay was also prepared for comparison. For the sand–clay mixtures, the sand and kaolin clay were sterilized before being added into the PVC cylinders. To ensure the sands and clays were thoroughly mixed, the premixing method was used (Table 1).

The density had an impact on the solidification effects [25]; therefore, all samples were prepared with an identical initial dry density (1.89 g/cm^3^). Because of the same total mass (300 g), the samples with a larger amounts of kaolin clay had a higher porosity. In the MICP or EICP treatment, the added kaolin clay expanded, leading to the decrease in porosity. This was why in the study of [16], the maximum mass percentage of added kaolin clays was only 7.5%. In the present study, the smaller space for solution to pass through allowed for a larger mass percentage of kaolin clays (10%).

#### 2.3.2. EICP Treatment

In the study of [14], three different MICP methods (injection, premixing and diffusion) were used to treat clayey sand. The clay percentages were controlled, and they drew the conclusion that different methods were suitable to treat clayey sands with different clay percentages. The maximum mass percentage of kaolin clay was 10% in the present study; therefore, the injection method was not suitable due to the low porosity of samples. The other two different methods, premixing and diffusion, were used to comparably investigate the solidification effect. It was noted that with the premixing method limited the treatment cycle of the urease solution and cementation solution. A total of 50 mL of urease solution (1000 U/L) and 50 mL of cementation solution were premixed with sand–clay mixtures. As for the diffusion method, the urease solution and of cementation solution with equal volume were mixed and then added into the PVC cylinders. In order to achieve a significant improvement, the urease solution and cementation solution were added with the diffusion method every two days. During the 12-day treatment period, the permeability of samples was measured every two days. The solidification test was conducted at 30 °C, and the mixture solution was set with an initial pH of 7.0.

#### 2.3.3. UCS and Content of CaCO_3_

After solidification, the samples were oven dried at 110 °C for 24 h before the unconfined compressive strength (UCS) tests. During the UCS tests, the loading speed was controlled at 1 mm/min. The added kaolin clays also provided a cementation effect; therefore, the untreated samples with different mass percentages of kaolin clay (2.5%, 5%, 7.5% and 10%) were prepared for comparison. The UCS results of these samples were obtained.

After the UCS test, the solidified specimens were divided into five parts along their length to measure the content of CaCO_3_ of each part. The content of CaCO_3_ was the ratio of the mass of produced CaCO_3_ precipitation to the mass of treated samples at this part. The clay soils contained metal and minerals, which might affect the results of CaCO_3_ contents measured by the acid pickling method. However, the contents of metal and minerals in clay soils were similar for samples. Therefore, the CaCO_3_ contents were comparable, and the acid pickling method was used in this study to obtain CaCO_3_ contents. Firstly, the samples were dried and weighed to obtain the total mass. After that, the samples were washed with 0.1 mol/L of HCl and then dried and weighed again. The difference of the two weights because of the acid leaching was the weight of the precipitated CaCO_3_. In addition, the solidification uniformity of EICP-treated sand–clay mixtures was evaluated by the contents of CaCO_3_ at different parts.

#### 2.3.4. Liquid Limit

The two different media, deionized water (κ′ = 80, $\sigma_{\mathrm{el}}$ < 5 μS/m, pH = 6.5) and 2M-NaCl solution (κ′ = 55, $\sigma_{\mathrm{el}}\;$ = 12 S/m, pH = 6.5), were used to measure the liquid limit of sand–clay mixtures via a fall cone test [26]. The pH and $\sigma_{\mathrm{el}}$ were measured with a pH/conductivity meter (S470-USP-K, Beiwei company, Shanghai, China). The values of κ′ of the fluids were obtained from [26,27].

#### 2.3.5. Scanning Electron Microscope Test

After the UCS test and CaCO_3_ content measurement, the sample D3 was subjected to a scanning electron microscope (SEM) test to obtain the microscopic characteristics. The SEM photo was obtained using the following apparatus: JSM-6300, JEOL company, Akishima, Japan.

## 3. Results and Discussions

### 3.1. Effect of the Ions in Kaolin Clay on Production of CaCO_3_

#### 3.1.1. Effect of Al_2_O_3_ on the Production Rate for CaCO_3_

In nature, CaCO_3_ crystals have three different types of crystal forms: calcite, vaterite and aragonite. Compared with the other two types of crystal forms, calcite is the most stable; vaterite is relatively unstable [28]. According to previous studies, the concentration of urease used in EICP would affect the type of CaCO_3_ crystals [29]. When the concentration of urease was lower, the CaCO_3_ crystals were mainly in the calcite form. Therefore, a lower urease concentration of 1000 U/L was chosen for the production of CaCO_3_ with the stable calcite form. In the kaolin clay used, silicon dioxide (SiO_2_) and Al_2_O_3_ are main ingredients. However, in nature, SiO_2_ is very stable [30,31]. Consequently, the effect of SiO_2_ on the production rate for CaCO_3_ was not considered in this study.

**Figure 1:**
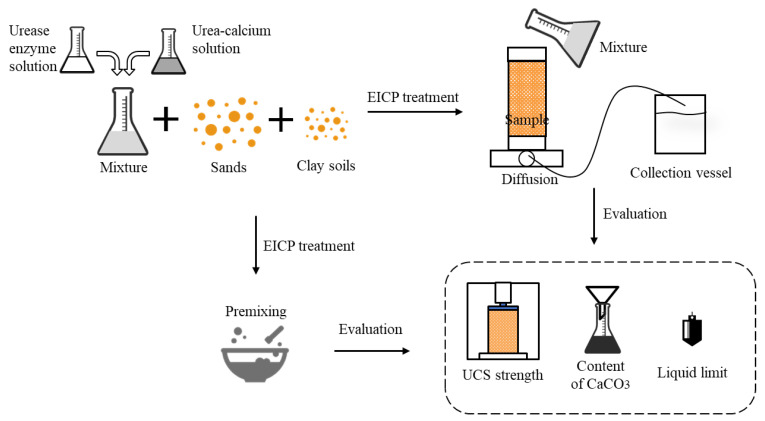
Schematic set-up of experiments.

The reaction period was only 48 h; different from the MICP reaction, urease for EICP was consumed continuously and no new urease was produced, and so the reaction time could not be any longer. Al_2_O_3_ was added to the urease solution to study its influence on the production rate for CaCO_3_ at different contents, as shown in Figure 1.

EICP is a complex biochemical process, and the production of CaCO_3_ precipitation depends on the concentration of calcium ions, dissolved inorganic carbon, and the pH of the solution [32]. The addition of Al_2_O_3_ did not have an impact on the production rate for CaCO_3_. The phenomenon was different from that for MICP [20,33]. It was because in MICP, the change of pH affected bacterial growth and urease activity, while it only had an effect on urease activity in EICP, which indicated there was a smaller inhabitation impact. The initial pH of the urease solution increased after being mixed with the cementation solution due to the reaction of Equation (3). The pH changed again after adding Al_2_O_3_, as shown in Equation (4). When adding 3 g of Al_2_O_3_, the pH decreased to around 7, which indicated that the pH was between 7 and 8.5 in the test; the impact of pH in this range could be ignored.

(3) $${\mathrm{CH}}_{3}{\mathrm{COO}}^{-}+H_{2}O⇄{\mathrm{CH}}_{3}\mathrm{COOH}+{\mathrm{OH}}^{-}$$

(4) $${\mathrm{Al}}_{2}O_{3}+3H_{2}O\to 2\mathrm{Al}{\left(\mathrm{OH}\right)}_{3}$$

#### 3.1.2. Effect of NaCl, MgCl_2_ and FeCl_3_ on the Production Rate for Calcium Carbonate

**Figure 2:**
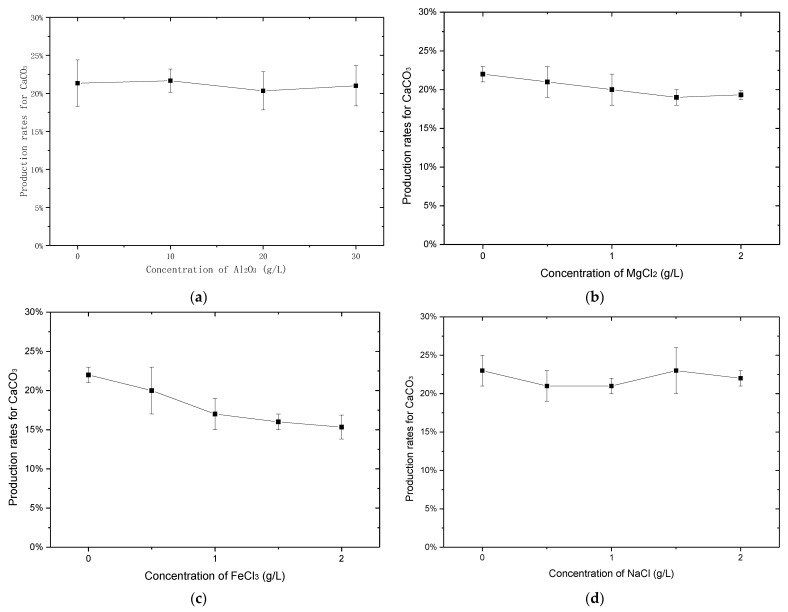
Effects of different compositions in kaolin clay on the production rate for calcium carbonate: (**a**) Al_2_O_3_; (**b**) MgCl_2_; (**c**) FeCl_3_; (**d**) NaCl.

Except for Al_2_O_3_, the effects of NaCl, MgCl_2_ and FeCl_3_ were also studied and the results are shown in Figure 2. However, the amounts of added NaCl, MgCl_2_ and FeCl_3_ were quite a bit smaller than the amount of Al_2_O_3_added. This was because the amounts of Na, Mg and Fe in the used kaolin clay were smaller. To obtain more credible results, the amount of added ions should be consistent with their contents in the used kaolin clay. From Figure 2, adding NaCl did not affect the production rate for CaCO_3_. Adding MgCl_2_ had a small impact on the production rate for CaCO_3_. The reason for this might be that adding MgCl_2_ could change the pH of solution, as shown in Equation (5).

(5) $${\mathrm{Mg}}^{2+}+2H_{2}O\to \mathrm{Mg}{\left(\mathrm{OH}\right)}_{2}+2H^{+}$$

Compared with NaCl and MgCl_2_, adding FeCl_3_ significantly decreased the production rate for CaCO_3_. With increased FeCl_3_, the production rate for CaCO_3_ almost decreased to 15%. This was because the hydrolysis reaction of FeCl_3_ resulted in more hydrogen ions than the hydrolysis reaction of MgCl_2_, which had a larger effect on the pH of solution due to the trivalent ion, as shown in Equation (6).

(6) $${\mathrm{Fe}}^{3+}+3H_{2}O\to \mathrm{Fe}{\left(\mathrm{OH}\right)}_{3}+3H^{+}$$

### 3.2. EICP Sand–Clay Mixtures Solidification in PVC Cylinders

#### 3.2.1. Permeability

The EICP method can be applied extensively because of the production of CaCO_3_ precipitation [34]. In contrast to MICP, the room for the production of CaCO_3_ precipitation can be smaller for EICP because no bacteria is used. Therefore, in the present study, the largest mass percentage of clay soils was 10%. In the study of [14], three different MICP methods (injection, premixing and diffusion) were used to treat clayey sand. They drew the conclusion that when the mass percentage of kaolin clay was larger than 7.5%, the space left in the sand–clay mixtures was not sufficient for the growth and reproduction of bacteria. In addition, it was hard to achieve multiple injections of the bacterial suspension and cementation solution. This was also why the maximum mass percentage of kaolin clay in Sun et al. [16] was 7.5%. Therefore, the injection method was not suitable due to the low porosity of samples.

**Figure 3:**
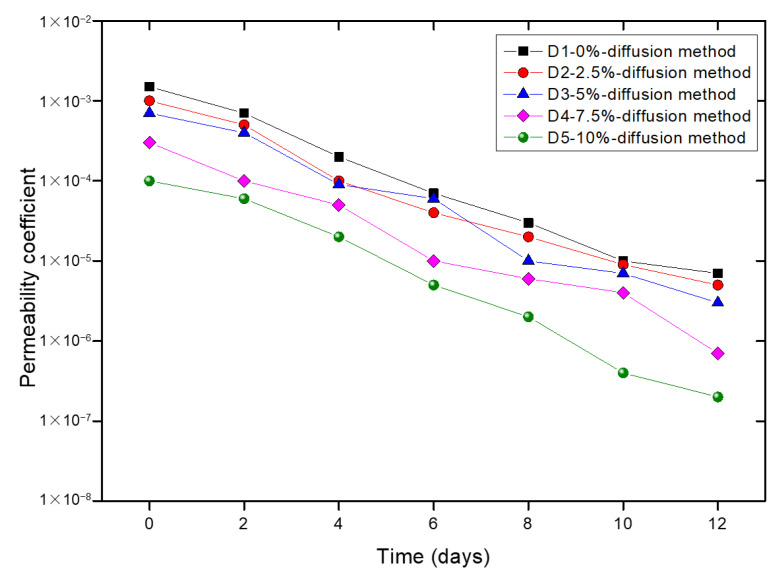
Change in the permeability of treated samples.

For MICP- or EICP-solidified samples, the property of permeation was an important indicator to evaluate the solidification effect. De Muynck et al. [35] assessed the durability from the permeation properties and resistance towards degradation processes. When the samples were solidified with the diffusion method, the decreasing ranges of permeability coefficients reached about 3–4 orders of magnitude for all samples (Figure 3). In the study of DeJong et al. [1], the MICP treatment resulted in an about 2–3 orders of magnitude of decreasing range for the permeability of silica. In this study, the decreasing range of permeability coefficients was larger because of the expansion of the added kaolin clays. Adding clay reduced the permeability, and the permeability coefficient became smaller with increased amounts of added clay soils. Therefore, the permeability coefficient of the sample without clay (D1) was always the largest. This was because clay soils expanded during the EICP treatment, decreasing the size of pores. Moreover, smaller pores made it easier for CaCO_3_ to remain rather than being flushed out; so, the sample D5 with the mass percentage of 10% added kaolin clays had the largest decreasing range of permeability coefficients. However, according to the study of [16], the sample with 2.5% kaolin clay had a larger decreasing range of permeability coefficients than the sample with 7.5% of kaolin clay. This was because in the MICP solidification test, smaller pores made little bacteria remain between particles, eventually leading to decreased contents of CaCO_3_; so, the decreasing range of permeability coefficients was smaller. However, with the EICP method, the smaller pores had a smaller impact on the production of CaCO_3_. Furthermore, adding more clay soils decreased bacterial urease activity and further inhibited the production of CaCO_3_ [16], which does not have to be considered in the application of EICP.

#### 3.2.2. UCS and Content of Precipitation

**Figure 4:**
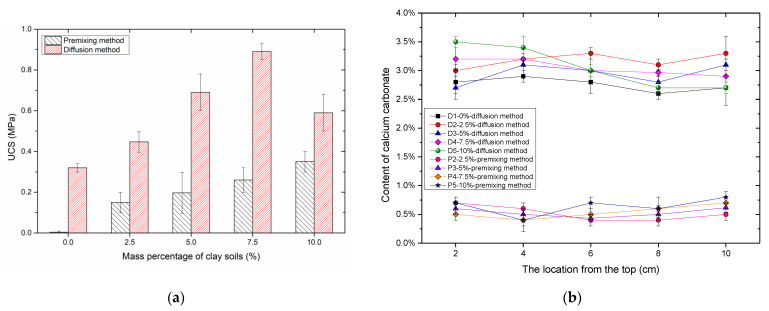
Comparison of treated samples with two different methods: (**a**) unconfined compressive strengths; (**b**) contents of calcium carbonate.

The strengths of samples made with the premixing method were much smaller (Figure 4a), because only one treatment cycle limited the amount of precipitated CaCO_3_ and the improvement of strength. Sample P5 had the highest strength (about 0.33 MPa). Moreover, sample P1 did not form a strong cemented unit. The difference between the strength of samples solidified with the two different methods demonstrated that the improvement of strength resulted from the precipitated CaCO_3_. The sample D4 had a larger strength than other samples. Small pores the cementation of CaCO_3_ and meant that it could remain; however, too much kaolin clay (10%) decreased the strength of the sand–clay mixture. The reason for this was that too much clay significantly decreased the initial porosity and the space left in samples was too small, eventually leading to a worse cementation homogeneity. In general, the amount of precipitated CaCO_3_ was similar for the samples with various mass percentages of clay soils. Therefore, the strength was contributed to by the cementing effect from clay soils and cementation homogeneity. The cementing effect from clay soils and better cementation homogeneity resulted in higher strengths than the results in [36,37].

The content of CaCO_3_ is also an important indicator for the evaluation of treatment effects [38]. Compared with the samples treated with the diffusion method, the amount of CaCO_3_ in the samples treated with the premixing method was much smaller (Figure 4b). The results were consistent with the results from UCS (Figure 4a). Moreover, the difference in the amount of CaCO_3_ was small at different location in samples, regardless of different treatment methods, which was much smaller than the difference in the amount of CaCO_3_ at different locations in [38]. There were two reasons: the first one was that MICP resulted in a worse cementing homogeneity than EICP; the second reason was due to the different calcium resource used in [38]. These are based on the study of [39] who reported that the mortar treated with Ca(CH_3_COO)_2_ had better solidification homogeneity than the samples treated with CaCl_2_ or Ca(NO_3_)_2_. For the samples with the diffusion method, the increasing mass percentages of added kaolin clay slightly affected the average contents of CaCO_3_. For D5, the content of CaCO_3_ at the top was larger than at the bottom, because adding too much kaolin clay made pore space smaller and the diffusion direction had a significant effect on the cementing homogeneity. With smaller pore spaces, a larger amount of CaCO_3_ was produced at the top, which clogged up the pores and made it harder for the fluid to flow through. However, the cementing homogeneity was still better than the sand columns solidified with the MICP method [16,38]. For the samples with a mass percentage of added clays below 7.5%, both the premixing method and the diffusion method could guarantee the cementing homogeneity.

#### 3.2.3. Soil Consistency

**Figure 5:**
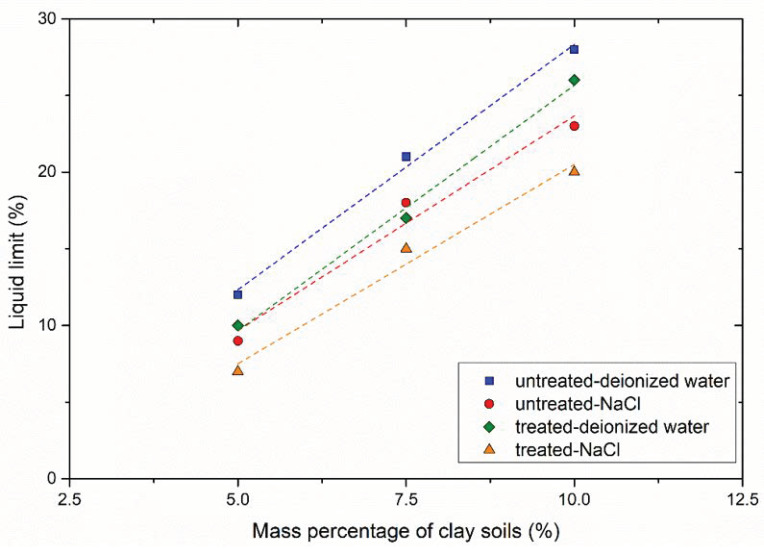
Liquid limit of samples: treated with the premixing method.

Higher clay contents showed higher LL values (Figure 5, which was similar to the results in [26]. For untreated samples, the counter-ions in the pore-fluid decreased the water adsorption of clay surfaces; therefore, the LL_DI_ was larger than the LL_Na_ [27]. The production of CaCO_3_ allowed for particle aggregation; so, both the values of LL_DI_ and LL_Na_ of treated samples were smaller than untreated samples. Moreover, the LL_DI_ of treated samples was also larger than LL_Na_. The reason for this might be that with the premixing method, the addition of solution slightly affected the LL values. The LL of treated sample P1 was not obtained because of a small mass percentage of clay soils.

**Figure 6:**
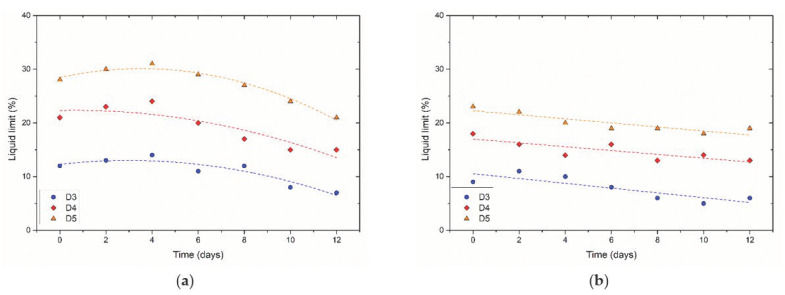
Change of liquid limit of samples treated with the diffusion method: (**a**) deionized water; (**b**) 2M-NaCl brine.

The LL_DI_ of sand–clay mixtures treated with the diffusion method increased first. It reached a peak point after two treatment cycles (four days), and then decreased to a constant value with the increasing treatment cycles (Figure 6a). The variation of LL_DI_ with time implies particle aggregation effects from EICP. Firstly, the addition of a mixed solution resulted in the increase in LL_DI_. All samples with different mass percentages of clay soils showed a peak LL_DI_. After that, EICP initiated particle aggregation via CaCO_3_ bonding, eventually decreasing the values of LL_DI_. The change in LL_DI_ seemed to be attributed to the equilibrium between the addition of solution and the simultaneous particle aggregation resulted from EICP. From Figure 6b, the LL_Na_ of samples with different mass percentages of clay soils gradually decreased. The EICP treatment cemented particles of sand–clay mixtures, thus altering the USCS classification. In the study of [26], the pore-fluid chemistry governed the LL of samples treated with xanthan gum in the brine. Similar to xanthan gum, the EICP treatment also increased the soil plasticity because of the addition of solution. Electrical sensitivity changed with the amount of precipitated CaCO_3_. Therefore, it is reasonable to assume that the pore-fluid chemistry governs the LL of EICP-treated samples in the brine.

#### 3.2.4. Scanning Electron Microscope Test

**Figure 7:**
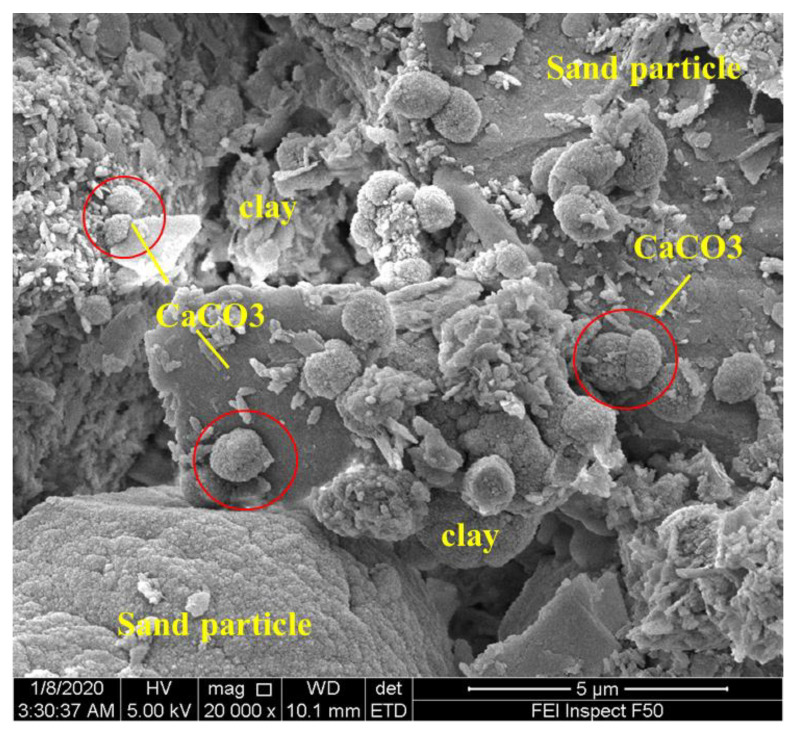
SEM image of treated sample D3, magnification = 20,000×.

The EDS test was not conducted in this study; however, previous studies have used the EDS test to confirm the CaCO_3_ produced in MICP-solidified clay soils [40,41,42]. SEM testing can be used to evaluate the treatment effect from a microscopic perspective. The sample D3 was subjected to an SEM test, as shown in Figure 7. In response to MICP treatment, a large number of CaCO_3_ crystals were produced between sand particles. Moreover, clay particles were coated by contacted CaCO_3_ crystals. In addition to their bridge function between sand particles, CaCO_3_ crystals were also deposited on the surface of sand particles. Furthermore, most CaCO_3_ crystals were vaterite, with a size of about 1–2 μm. In addition to spherical crystals, few amorphous crystals were found.

#### 3.2.5. Applications and Limitations

The strengths of sand–clay mixtures solidified with the diffusion method were much larger than the samples solidified with the premixing method, even reaching 0.9 MPa. The achieved strength with the diffusion method was more adequate for real-field applications. However, the optimum solidification conditions (e.g., reagent concentrations, urease activity, treatment cycles) of the two different methods still should be further studied for the sand–clay mixture solidification.

## 4. Conclusions

In this present study, EICP was used for the improvement of sand–clay mixtures. The impact of the ions used in kaolin clays on the production rate of CaCO_3_ was studied via the calcification test. The solidification test was conducted using the two different methods, and the solidification effect of the EICP method was evaluated. Moreover, the two types of liquid limits of treated samples were measured with deionized water and 2M-NaCl brine, respectively. Several conclusions were obtained:

(1) The addition of Al_2_O_3_, NaCl and MgCl_2_ had slight impact on the production rate for CaCO_3_, while the addition of FeCl_3_ significantly inhibited the production of CaCO_3_.

(2) The permeability coefficients decreased by about 3–4 orders of magnitude for all EICP-treated samples when the diffusion method was used for treatment. For samples with 10% kaolin clay, the change in permeability was the largest.

(3) With the premixing method, the sample with 10% clay had the largest strength, while the sample with 7.5% kaolin clay and solidified with the diffusion method had a higher strength than other samples with different mass percentages of clay.

(4) In contrast to injecting commonly used materials (e.g., cement, lime) to improve the properties of soils, the MICP or EICP technique is environmentally friendly and would not cause a negative influence on environment. Moreover, compared with MICP, EICP can guarantee cementing uniformity. With more kaolin clay, the cementing uniformity of a sample solidified with the diffusion method was relatively worse; however, the improvement was better than that solidified with the premixing method.

(5) Both the LL_DI_ and LL_Na_ of samples treated with the premixing method decreased with increasing mass percentage of clay soils. However, the LL_DI_ of samples treated with the diffusion method increased first, and then decreased with increasing treatment cycles. Moreover, the LL_Na_ of samples treated with the diffusion method decreased, regardless of different mass percentages of clay soils. Different from the deionized water, the pore-fluid chemistry had a larger effect on the liquid limit with 2M-NaCl brine.

## Figures and Tables

**Table 1:** Sample arrangement for tests.

Sample No.	Mass Percentages Of Kaolin Clay	Mass of Sands (g)	Mass of Clay Soils (g)	Treatment Cycle	Treatment State
U1	0%	300	0	/	Untreated
P1		300	0	1	Premixing treated
D1		300	0	6	Diffusion treated
U2	2.5%	292.5	7.5	/	Untreated
P2		292.5	7.5	1	Premixing treated
D2		292.5	7.5	6	Diffusion treated
U3	5%	285	15	/	Untreated
P3		285	15	1	Premixing treated
D3		285	15	6	Diffusion treated
U4	7.5%	277.5	22.5	/	Untreated
P4		277.5	22.5	1	Premixing treated
D4		277.5	22.5	6	Diffusion treated
U5	10%	270	30	/	Untreated
P5		270	30	1	Premixing treated
D5		270	30	6	Diffusion treated

## Data Availability

The data presented in this study are available on request from the corresponding author.
